# Childhood bone tumours in primary care: helping GPs to identify ‘the needle in the haystack’

**DOI:** 10.3399/bjgp23X734673

**Published:** 2023-07-28

**Authors:** Dhurgshaarna Shanmugavadivel, Jo-Fen Liu, Ashley Ball-Gamble, Angela Polanco, Kavita Vedhara, Paul Nathan, David Walker, Shalini Ojha

**Affiliations:** Academic Unit of Population and Lifespan Sciences, School of Medicine, University of Nottingham. Nottingham.; Academic Unit of Population and Lifespan Sciences, School of Medicine, University of Nottingham. Nottingham.; Children’s Cancer and Leukaemia Group, Leicester.; Children’s Cancer and Leukaemia Group, Leicester.; Academic Unit of Population and Lifespan Sciences, School of Medicine, University of Nottingham. Nottingham.; Hollybrook Medical Centre, Parkfields Surgery, Littleover, Derby.; Children’s Brain Tumour Research Centre, School of Medicine, University of Nottingham, Nottingham.; Academic Unit of Population and Lifespan Sciences, School of Health Sciences, University of Nottingham, Nottingham, and Children’s Hospital, University Hospitals of Derby and Burton NHS Trust, Derby.

## INTRODUCTION

In 2018, the World Health Organization declared childhood cancer as a global disease burden, launching a Global Initiative to improve survival to 60% worldwide by 2030.^1^ If achieved, it is estimated that an extra 1 million children’s lives will be saved. In the UK, childhood cancer is the largest illness cause of death in childhood in 1–19-year-olds and the incidence continues to rise.^2^ Unlike in adult cancers, there are no modifiable risk factors or cost-effective screening options and so early diagnosis is key to reducing morbidity, mortality, and late effects from treatment burden.

## THE DIAGNOSTIC JOURNEY

The adult cancer diagnostic journey has been defined in the literature as a combination of many time intervals including the time from the first symptoms to presentation and then from presentation to diagnosis.^3^ Children and young people (CYP) with cancer experience prolonged and clinically significant intervals throughout the health service both at primary and secondary care level. In bone tumours, the time to diagnosis can be life and limb saving, reducing the need for amputation.

The national HeadSmart campaign aimed to address diagnostic delay for childhood brain tumours in the UK by developing gold-standard clinical guidance and disseminating it through a public and professional awareness campaign.^4^ This was associated with halving the time to diagnosis from 14.4 weeks to 6.7 weeks (median) in 5 years. The time from presentation to diagnosis was shortened from 3.3 weeks to 1 week.^4^ Based upon this success, the model is being replicated for bone and abdominal tumours, where diagnostic delay is of concern and survival estimates are poorer compared with European counterparts.^5^^,^^6^

This article covers the clinical practice implications for bone tumours in children.

## THE CHALLENGES OF A BONE TUMOUR DIAGNOSIS

### Non-specificity of symptoms

CYP with bone tumours present with non-specific symptoms that can be attributed to many other, more common illnesses and injuries.

A systematic review and meta-analysis were conducted to identify how bone tumours present in this cohort. This identified 29 bone tumour symptoms/signs.^7^^,^^8^ The top symptoms, ranked by pooled proportions, were bone pain (76%), swelling (21%), fever (4%), history of trauma (3%), functional limitation (3%), palpable mass (3%), pain and swelling (2%), limp (2%), and pathological fracture (2%).

These symptoms are seen daily in CYP within primary care, and identifying those who need further investigation can be difficult.

### Perceived rarity and lack of awareness

There is a misconception that childhood cancer is rare, despite it being the leading illness cause of child death in >1-year-olds. An individual’s cumulative risk of cancer from birth to age 15 years is 1 in 450, with 1840 new cases diagnosed in CYP aged 0‒15 years each year in the UK.^2^^,^^9^

As a result, public and professional awareness of childhood cancer is low. A face-to-face public survey (*n* = 1000) showed that the general public are unaware of childhood cancer risk, have a lack of confidence in recognising signs and symptoms, and have inaccurate knowledge of which symptoms could be caused by cancer in children.^10^ Bone tumour symptom awareness was particularly low with considerably fewer than half of responders being aware of presenting symptoms of recurrent/persistent bone pain (23%), bone or joint swelling (27%), and slow recovery after injury (14%) as typical symptoms of malignancy.

This combination of perceived rarity and a lack of awareness contributes to lengthier diagnostic intervals. Qualitative research with parents who have experienced a childhood cancer diagnosis highlights that cancer was never on their radar prior to diagnosis and that healthcare professionals who saw them had not considered it as a diagnosis either.^11^

## DEVELOPING A CLINICAL GUIDELINE WITH EXPERT PRIMARY CARE OPINIONS

A Delphi consensus process was conducted to use professional expertise from all specialties who see CYP to incorporate the evidence from systematic reviews into a clinical guideline.^12^ This consensus process involved 133 healthcare professionals, including 57 GPs. Consensus was reached on 64 statements through 2 rounds of Delphi, which will form the backbone of the new clinical guideline.

The statements were split into categories:

- Best practice in conducting the consultation: referral, imaging, predisposing factors;
- Bone tumours: general, bone pain, swelling, mass/lump, restricted movement/limp; and
- Abdominal tumours: general, abdominal pain, abdominal mass, haematuria, abdominal distension.

**Figure 1.**
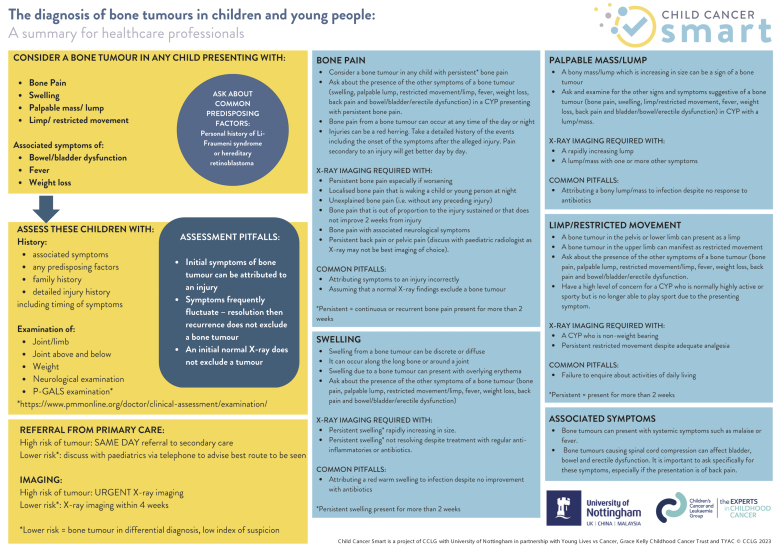
*Summary of bone tumour recommendations. Reproduced with permission from CCLG.*

The key statements have been summarised in Figure 1.

## HOW CAN GPS USE THIS TO IDENTIFY ‘THE NEEDLE IN THE HAYSTACK’?

While these provide detailed guidance, here are some general principles that can aid prompt diagnosis:

### Think childhood cancer

‘Three strikes’ with the same complaint without a diagnosis should justify considering cancer as a potential differential.^9^ A high index of suspicion should be maintained for bone tumours in children presenting with persistent or concerning symptoms. GPs should use the symptom checklist (Figure 1) in their consultations to identify symptoms and ask about duration. This will identify those who require further investigation. Persistent is defined as occurring on most days for 2 weeks. *Two or more persistent symptoms require imaging*. Imaging requests should be labelled as urgent and carried out within 24 hours; however, an imaging request should not delay referral.

### Phone a friend

*If you have a suspicion of cancer, call your local paediatrician for discussion*. Often, this will ensure that the CYP is seen in the most suitable place according to need, either the same day or in rapid-access or standard clinic settings. Discussing the case, rather than sending a written referral, provides the opportunity for a more nuanced discussion about probability, allowing more prompt and shared decision making.

### Address the elephant in the room

Parents and families who attend recurrently with the same symptoms often have a gut instinct that something is wrong, just as clinicians do. GPs should ask them directly, *‘What are you worried this could be?’* This allows honest and genuine discussion about their concerns, and whether they can either be reassured, reviewed, or referred for further investigations.

### Avoid the injury red herring

It is common for CYP presenting with bone symptoms to have some recall bias when asked if they have injured themselves, and so it is important to take a thorough injury history including mechanism, onset of symptoms, and any relieving factors. If symptoms are a result of an injury, it should have improved considerably within 2 weeks. *If no or slow improvement after 2 weeks, they need an X-ray*.

## CONCLUSION

Bone tumours can cause a diagnostic dilemma for primary and secondary care clinicians. Clear and concise tumour-specific guidance can empower GPs with the confidence and knowledge to assess and investigate those CYP who need it in a prompt manner. The full clinical guideline is due to be published later in 2023, with messaging amplified through a new childhood cancer awareness campaign, called Child Cancer Smart.
